# Lower plasma insulin levels during overnight closed-loop in school children with type 1 diabetes: Potential advantage? A randomized cross-over trial

**DOI:** 10.1371/journal.pone.0212013

**Published:** 2019-03-08

**Authors:** Ulrike Schierloh, Malgorzata E. Wilinska, Ineke M. Pit-ten Cate, Petra Baumann, Roman Hovorka, Carine De Beaufort, Spidiman Study Group: Single Port Insulin Infusion for Improved Diabetes Management

**Affiliations:** 1 Pediatric Clinic, Centre Hospitalier de Luxembourg, Luxembourg, GD de Luxembourg; 2 Department of Paediatrics, University of Cambridge, Cambridge, United Kingdom; 3 Wellcome Trust-MRC Institute of Metabolic Science, University of Cambridge, Cambridge, United Kingdom; 4 Faculty of Language and Literature, Humanities, Arts and Education, University of Luxembourg, Luxembourg, GD de Luxembourg; 5 Joanneum Research Forschungsgesellschaft, Graz, Austria; 6 Department of Pediatrics, University Hospital Brussels, Brussels, Belgium; Universidad Miguel Hernandez de Elche, SPAIN

## Abstract

**Background:**

Studies have shown that overnight closed-loop insulin delivery can improve glucose control and reduce the risk of hypoglycemia and hence may improve metabolic outcomes and reduce burden for children with type 1 diabetes and their families. However, research so far has not reported insulin levels while comparing closed-loop to open-loop insulin delivery in children. Therefore, in this study we obtained glucose levels as well as plasma insulin levels in children with type 1 diabetes to evaluate the efficacy of a model—based closed-loop algorithm compared to an open-loop administration.

**Methods:**

Fifteen children with type 1 diabetes, 6–12 years, participated in this open-label single center study. We used a randomized cross over design in which we compared overnight closed-loop insulin delivery with sensor augmented pump therapy for two nights in both the hospital and at home (i.e., 1 night in-patient stay and at home per treatment condition). Only during the in-patient stay, hourly plasma insulin and blood glucose levels were assessed and are reported in this paper.

**Results:**

Results of paired sample *t*-tests revealed that although plasma insulin levels were significantly lower during the closed-loop than in the open-loop (Mean difference 36.51 pmol/l; *t*(13) = 2.13, *p* = .03, effect size *d* = 0.57), blood glucose levels did not vary between conditions (mean difference 0.76 mmol/l; *t*(13) = 1.24, *p* = .12, *d* = 0.37). The administered dose of insulin was significantly lower during the closed-loop compared with the open-loop *(*mean difference 0.10 UI; *t*(12) = 2.45, *p* = .02, *d* = 0.68).

**Conclusions:**

Lower insulin doses were delivered in the closed-loop, resulting in lower plasma insulin levels, whereby glucose levels were not affected negatively. This suggests that the closed-loop administration is better targeted and hence could be more effective.

## Introduction

Patients with type1 diabetes need lifelong insulin treatment and a good metabolic control to prevent long-term complications [1]. Recent reports suggest that this good control is essential from an early age onwards [2,3] however; most young people with type1 diabetes do not meet the treatment targets [4]. The day-to-day management to achieve a good metabolic control is challenging for the child and its family and has a major impact on their quality of life [5,6]. Therefore, optimizing metabolic control in children with type 1 diabetes has led to intensified research in improved pump technology and the use of sensor augmented pump [7,8]. Technical developments in closing the loop between the glucose sensor data and insulin administration (so called „artificial pancreas“) should improve metabolic outcome and reduce burden for the pediatric patient and his family. Several studies have shown that overnight closed-loop insulin delivery can improve glucose control and reduce the risk of hypoglycemia in young patients with type 1 diabetes [9,10,11]. As a more targeted insulin administration has been associated with lower insulin doses and plasma insulin levels in adolescents [12,13] to date only scarce data is available for a pediatric population [14]. Insulin sensitivity differs between different age groups [15,16] and requests a validation of algorithms developed for adolescents and adults also in children. Therefore, in this exploratory study we compared overnight insulin and glucose profiles in children with type 1 diabetes, who received in random order either the sensor augmented pump (pre programmed–hourly- basal insulin rate, but with continuous glucose measurements, visualized on the insulin pump) or the closed-loop insulin delivery system, with a model-based algorithm, (variable -15 min- basal insulin rate, steered by the algorithm based on continuous glucose measurements).

## Methods

The national Luxemburgish ethics committee (Comité national d’Ethique de Recherche Luxembourg) approved this specific study (on November 12^th^, 2013) before we started it.

The authors confirm that all ongoing and related trials for this intervention are registered.

Although the CHL (Centre Hospitalier de Luxembourg) was already recognised by ClinicalTrials.gov., local administrative procedures have taken more time than initially expected. As the start of the study, and inclusion of the first patients during their holidays, was already planned, we did proceed with the study, despite the ongoing administrative challenges. Once solved, the entry in the registry was introduced.on March 28th, 2014.

Clinical Trials.gov Identifier: NCT 02099409

## Participants and design

**Fig 1:**
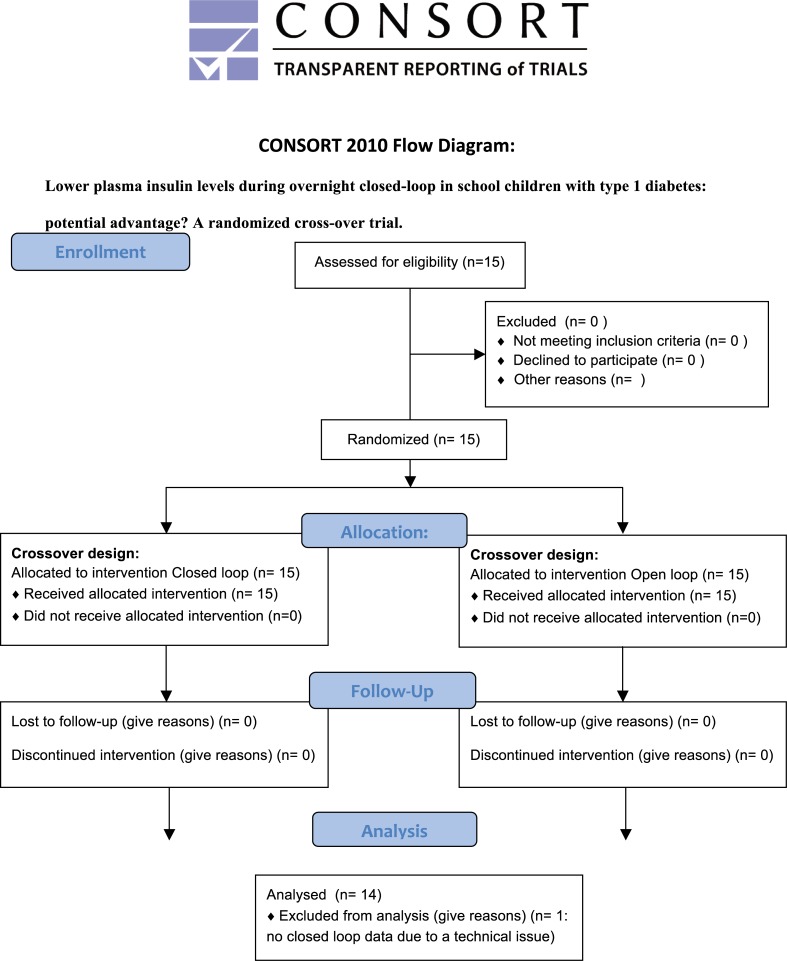
Consort 2010 flow diagram.

Fig 1 shows the workflow of this study.

### Participants

Fifteen children (8 males) were enrolled in the study.

All the participants fulfilled the inclusion criteria: age between 6 and 12 years, type 1 diabetes for at least 6 months, on insulin pump treatment for at least 6 months and HbA1c below 11% (below 96.72 mmol/mol).

Exclusion criteria for participation in the study were another type of diabetes than type 1, physical or psychological disease likely to interfere with an appropriate conduct of the study and current drug therapy knowing to interfere with glucose metabolism.

The participating children had no physical or psychological disease interfering with an appropriate conduct of the study. Prior to enrolment, written informed consent was obtained from the parents and all children gave their informed assent.

The children were enrolled between December 2013 and July 2014. The study was completed in July 2014.

### Study design

In this open-label, single center, randomized cross-over study in the Children’s Hospital in Luxemburg, all participants received treatment in a randomly assigned order including open-loop and closed-loop insulin administration during two 1-night in-patient stays (CRC). The Florence D2 closed-loop (CL) system (University of Cambridge, UK) integrates data from the sensor to steer the insulin administration based on a model predictive control algorithm [17] during the closed-loop nights. More specifically, every 15 minutes the continuous glucose monitor (FreeStyle Navigator II, Abbott Diabetes Care, Alameda, CA) transmitted the real time glucose values to the laptop running control algorithm, which in turn instructed the insulin pump (Dana R, SOOIL, Seoul, South Korea) to deliver the calculated insulin dose. During the study a second continuous glucose measurement device (Dexcom G4, Dexcom, San Diego, CA) has been used to obtain independent glucose levels, which was not integrated in the closed loop system. Blood samples were collected to measure plasma insulin and glucose levels.

The open-loop condition (OL) was similar to the closed-loop except for the use of the FLORENCE D2. In the open-loop, the sensor for continuous glucose monitoring worked independently from insulin administration, and insulin was delivered at a pre-programmed basal rate. All patients received insulin Aspart during the study period.

Before the trial, a specialist nurse provided participants and their parents training on diabetes management and device use and had their knowledge assessed by a ten-question multiple choice test concerning pump therapy and general diabetes understanding. During a run-in period of 2 to 3 weeks the patients used the study insulin pump and the continuous glucose monitoring to get experience with the devices.

The patients arrived in the clinic in the late afternoon of day 1 of the study. At arrival a venous cannula was inserted in the antecubital vein after applying anesthetic cream. Venous plasma insulin levels (pmol/l) were measured by an immunochemiluminometric assay (Invitron, Monmouth, UK) intra assay CV 4.7%, inter assay CV 7.2–8.1%. Venous whole blood glucose was measured using the I-Stat (glucose oxidase method ABBOTT) reporting a CV at 41 mg/dl of 1.6% and 0.8% in the higher range (289 mg/dl).

Participating children received a pre-defined dinner (mixed-meal), with calculated 50 g of carbohydrates, at 7 pm. There was no snack served after this meal. Bolus calculation was based on pre-programmed bolus advice. The patients were randomly divided into two groups (Table 1). Patients in group one were assigned to start in the closed-loop condition, patients in group two started in the open-loop. In the closed-loop condition, the Florence D2 system was started after dinner, whereas in the open-loop patients continued their insulin treatment with pre-programmed basal rate. Overnight, during the hospital stay hourly blood samples for glucose and insulin levels were taken for all patients. The next morning (day 2) before breakfast, the closed-loop control was stopped and the patients returned to their usual insulin treatment with the study devices and were discharged from hospital. The following night home data were collected. More specifically, the closed loop system was restarted at home at the evening after dinner. In the open loop, patients continued their usual therapy both during the inpatient stay and at home. In both conditions we collected sensor data. After each study night, sensor and pump data were downloaded, anonymised and used for data analysis.

**Table 1:** Demographic characteristics of the participants (N = 14).

Variable	Mean	SD	Range
Age (years)	10.38	2.12	6.40–12.90
BMI (kg/m^2^)	19.07	3.60	13.70–29.20
HbA1C (%)	7.91	1.02	6.90–10.60
Duration Diabetes (years)	5.82	2.54	1.80–9.90
Pump use (years)	5.22	2.35	1.50–8.80
Total daily insulin per bodyweight (UI/kg/day)	0.82	0.25	0.52–1.35

**Fig 2:**
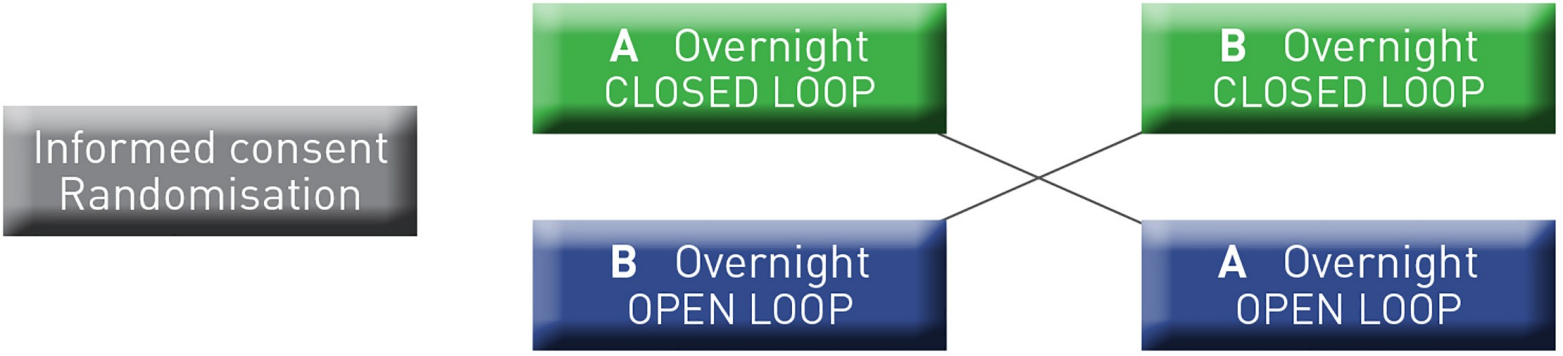
Cross-over design of the study.

The cross-over design of the stud. Is illustrated in Fig 2.

### Study outcomes and statistical analysis

The study was an exploratory study, including two overnight hospitalisations with intravenous blood sampling and the use of three devices (2 glucose monitoring devices and 1 insulin pump), in children. We did not conduct an a-priori power analysis as this study mainly set out to test the feasibility of the use of closed loop paradigm versus traditional insulin administration in young children. However, based on previous research [14] we expected that children in the closed loop would spend on average 20% more time (SD = 25) in the target glucose range than children in the open loop. We therefore aimed to include 12 children in our analysis, which would provide 83% power [18] to detect such increase (α = 0.05, one tailed). Allowing for a 20% drop out we therefore initially recruited 15 participants.

Study outcomes included the administered insulin dose and plasma insulin and glucose levels during closed and open loop nights as well as time spent below and above the target glucose level. Night was defined as time between 11pm and 7am. Results are expressed as mean ± 1 standard deviation.

For each of the three outcome measures we conducted paired samples *t*-tests, whereby for each child average overnight administered insulin dose, plasma insulin and glucose levels in the closed loop were compared with levels in the open loop. Before conducting our analyses we checked the distributions of the difference scores using the Shapiro Wilks test of normality [19] applying a conservative alpha level (p < .01) given the small sample [20]. For all three distributions, the Shapiro Wilks test was not significant, hence parametric tests were applied. We conducted paired sample *t*-test for the average administered insulin dose and plasma insulin and glucose levels, as we were interested in insulin delivery and plasma insulin and glucose levels throughout the night rather than at specific points during the night. For each comparison, we also computed the effect size *d*, reflecting the mean difference between the observations in the open and closed loops in units of standard deviation. The time spent in target glucose range from 3.9 mmol/l to 8.0 mmol/l based on a secondary CGM (Dexcom)–was analysed using a mixed Poison model (with offset).

We used IBM SPSS Statistics for Windows version 24, IBM Corp. Released 2016. IBM SPSS Statistics for Windows, Version 24.0. Armonk, NY: IBM Corp.

## Results

For one patient we did not have closed-loop data due to a technical issue. Therefore, results include data of 14 patients (demographic characteristics in Table 1).

### Insulin administration during the night

Descriptive statistics for insulin dose are presented in Table 2. One patient had to be excluded from this analysis due to incomplete insulin samples. During the night the administered dose of insulin was significantly lower during the closed-loop compared with the open-loop (0.5 ± 0.3 U/kg/day versus 0.6 ± 0.3 U/kg/day; *t*(12) = 2.45; *p* = .02, effect size *d* = 0.68). More specifically, the mean difference for the insulin dose administered during the closed-loop and open-loop was 0.10 UI (95% confidence interval 0.01–0.19 UI).

**Table 2:** Descriptive statistics for the average insulin administration dose and plasma insulin and glucose levels in open- and closed-loop (N = 14).

	Open-Loop		Closed-Loop		
	Mean	SD	Mean	SD	95% Confidence Interval of the difference
Insulin dose (UI)	0.6	0.3	0.5	0.3	0.0. . . . . . . .0.2
Plasma insulin level (pmol/l)	233.9	169.7	197.4	167.6	-0.5. . . . . . .73.5
Glucose concentration (mmol/l)	7.4	1.3	8.2	2.2	-2.1. . . . . . .0.6
Glucose in target range (% time)	53.2	21.5	57.1	27.5	-16.3. . . . .8.3

Results of a 2×9 mixed method analysis with treatment (open vs. closed loop) as between group factor and insulin levels (9 measurements throughout the night) revealed significant main effects of treatment, F(1,13) = 4.58, p = .03, one sided, η^2^ = .26) and insulin levels, F(8,104) = 12.72, p < .001, η^2^ = .49). These results indicated that during closed loop the mean insulin level was lower than in the open loop. Furthermore, insulin levels fluctuated throughout the night. The interaction effect treatment × insulin was not significant (F(8,104) = 0.21, p = .99, η^2^ = .02, indicating that the fluctuations in insulin were similar in both treatment conditions.

### Plasma insulin levels during the night

**Fig 3:**
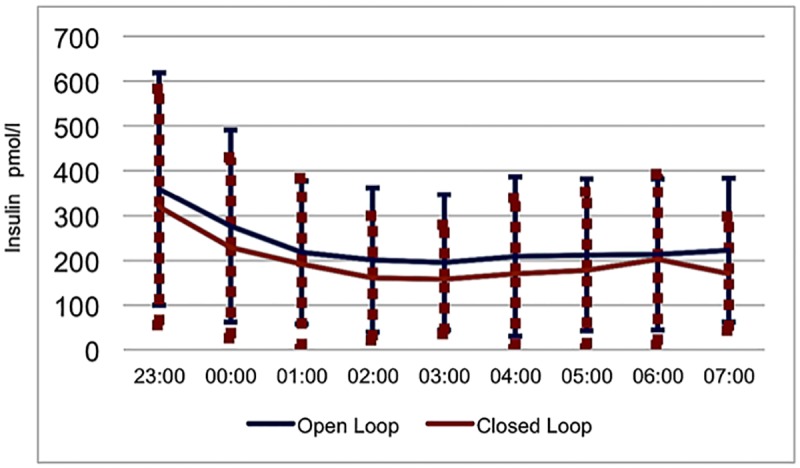
Overnight insulin levels (mean ± (SD) Standard Deviation) in children, treated either with open loop (sensor augmented pump) or closed loop.

As illustrated in Fig 3 plasma insulin levels were significantly lower during the closed-loop than during the open-loop for all children (197.4 ± 167.6 pmol/l versus 233.9 ± 169.7 pmol/l *t*(13) = 2.13, *p* = .03, *d* = 0.57). For descriptive statistics see Table 3.

**Table 3:** Hyper- and hypoglycemic events during the night in open and closed loop.

	Open loop	Closed loop	Total
Glucose < 3.3 mmol/l	4	0	4
3.3mmol/l ≤ glucose ≤ 10mmol/l	105	100	205
Glucose >10mmol/l	17	26	43
	126	126	252

### Plasma glucose levels during the night

Glucose levels did not differ between open- and closed-loops (8.2 ±1.2 mmol/l versus 7.4 ± 1.3 mmol/l; t(13) = 1.24, p = .12, d = 0.37).

More specifically, mean glucose levels did not vary as a function of insulin dose administration. However, to control for difference in insulin dose between the two treatment loops, we conducted a repeated measures ANOVA with mean blood glucose levels as within subjects factor, and entered the mean difference of insulin dose as a covariate. Results showed that after controlling for the mean difference of insulin dose, glucose levels did vary by treatment condition, F(1,11) = 12.43, p = .005, η^2^ = .53, indicating that the adjusted glucose levels in the closed loop (M = 8.18, SD = 2.30) were higher than in the open loop (M = 7.42, SD = 1.37). Furthermore the glucose × insulin dose interaction was significant, F(1,11) = 13.93, p = .003, η^2^ = .56, indicating that insulin dose was lower in closed versus open loop, whereas glucose levels were slightly higher.

### Time spent in target glucose target range from 3.9 mmol/l to 8.0 mmol/l based on the independent CGM (Dexcom G4)

No severe hypoglycemia or hyperglycemia or serious adverse events were seen in closed-loop or in open-loop during the study period. In the hospital setting, no significant difference was found in time spent in target range during closed-loop versus open-loop (observed percentage time in glucose target with closed loop 57.1 ± 27.5% versus open-loop 53.2 ± 21.5%).

Looking more closely at the glucose data, some instances of glucose levels of below 3.3mmol/l (3.2% in OL vs 0.0% in CL) or over 10mmol/l (13.5% in OL and 20.1% in CL) were measured (see Table 3).

We used a Chi-square analysis to investigate the relationship between treatment condition and glucose levels within or outside range. Results of the Chi-square analyses revealed that glucose levels were unrelated to treatment loop (Chi-square = 0.65, p = .42; Fisher exact = 0.52, not significant), indicating that there were no systematic variations in glucose levels as a function of treatment (open vs. closed loop).

## Discussion

During the inpatient stay, insulin dose administration and plasma insulin levels during the closed-loop were significantly lower than in the open-loop without affecting glucose levels. Most studies, demonstrating the positive effect of closed-loop treatment in patients with diabetes, are focusing on the glucose time in target or HbA1c levels. In this feasibility study, we included not only glucose parameters, but also insulin doses and plasma insulin levels as important markers for a more physiological insulin administration. The initial closed loop studies in youth suggest as well a potential impact on the plasma insulin concentration, which did not reach significance [14]. Although achievement of normoglycemia is one of the primary objectives in children with type 1 diabetes, intensive insulin treatment is often associated with higher insulin doses and an increase in weight and obesity [21–23]. The long term risks of hyperinsulinemia in obesity and type 2 diabetes is widely debated, its presence and potentially long term impact on the outcome in type 1 diabetes patients is less frequently discussed [24,25]. Adding a glucose lowering and insulin-sensitizing drug, such as metformin, has not shown a positive benefit/risk ratio, indicating that prevention of overweight, obesity is needed [26].

It should be noted that after controlling for differences in insulin dose between the two treatment arms, glucose levels in closed loop were higher than in open loop. Although blood glucose levels were higher in closed loop, no systematic differences in blood glucose levels (in- versus outside target range) as a function of treatment loop were found. A more physiological insulin replacement through closed loop may therefore help to prevent hyperinsulinemia and result in a lower insulin dose although more and longer observations will be needed to confirm this.

In sum, in this feasibility study lower insulin doses and plasma insulin levels during the nights in closed-loop suggest a better-targeted insulin administration without negatively impacting glucose levels.

### Limitations of this study

The number of patients is small and the duration of the study short. However, results of this cross over study, clearly suggests a more targeted insulin dosing associated with lower insulin levels. Future studies could focus on long-term effects and differences between different age groups and settings (e.g. at home). Even if time in glucose target is still the main objective of the treatment of children with type 1 diabetes, the demonstrated reduction of insulin levels may contribute as well to improve long-term outcome.

## Supporting information

S1 Checklist CONSORT 2010 checklist. (DOC)

S1 Protocol SPIDIMAN 01 protocol. (DOC)
