# Supplementary material for: Lower plasma insulin levels during overnight closed-loop in school children with type 1 diabetes: Potential advantage? A randomized cross-over trial
Source: PLoS One. 2019 Mar 8;14(3):e0212013. doi: 10.1371/journal.pone.0212013 (PMC6408001; doi:10.1371/journal.pone.0212013)
Supplement: S1 Protocol — (DOC) [file pone.0212013.s002.doc]

**This is the original document submitted to our national ethics committee before we started this specific study.**

**SPIDIMAN 01 protocol**

Synopsis

Title

Open label single centre randomized cross over study

to validate current algorithms and evaluate safety and efficacy of closed loop insulin delivery (FlorenceD2 system) in children with type 1 diabetes between 6 – 12 years of age

Objectives:

Long Term

Improve and facilitate diabetes management in children with

type 1 diabetes by closing the loop

Short term

- Validation of the algorithms for the overnight closed loop system in children with t1dm between 6-12 y in the clinic and at home. Collect glucose, insulin and insulin infusion rate data, helping to improve future ability of computer–based closed loop algorithms to control blood glucose in children with type 1 diabetes

Primary outcome

- Time spent in normal glucose range (70-144 mg/dl) of the children with type 1 diabetes 6-12 years with the use of an automated overnight closed loop pump uploaded with sensor and automated insulin feedback system without manual intervention overnight in the clinic and at home setting as measured by secondary CGM (start 1800, post dinner bolus, and till pre breakfast bolus 0730),

Secondary outcome (secondary CGM)

- Glucose variability (excursions) when treated with overnight closed loop in the clinic and at home (1800-0730)
- Time spent in hypoglycaemia (<60 mg/dl), as measured by the sensor, in the clinic and at home
- Time spent in hypoglycaemia (<70 mg/dl), as measured by the sensor, in the clinic and at home
- Time spent in hyperglycaemia (>180mg/dl,), as measured by the sensor, in the clinic and at home
- Comparison of the performance of two sensors at different site

Exploratory

- - User evaluation of the overnight closed loop.
  - Gut flora impact on glycaemic variability

Efficacy endpoint:

- Time spent with glucose values between 70-144 mg/dl (sensor measurements by secondary sensor) comparing the nights with and without automated insulin delivery (FlorenceD2 system with closed loop)
- Time spent in normoglycaemia (70-144 mg/ml as observed by blood glucose measurements) comparing the nights with and without automated insulin delivery

Safety endpoint

- Reduction in Time spent in hypoglycaemia as observed by blood glucose measurements during closed versus non closed loop
- Time spent in low glucose measurements (sensors, <60 mg/dl)

and/or perceived hypoglycaemic values during closed and non closed loop in the clinic and at home

Sample size: 12 children completed, 15 to control for potential drop outs and incomplete read-outs.

Inclusion criteria

Age 6- 12 years, with type 1 diabetes mellitus for at least 6 months, with at least CSII for 6 months.

HbA1c <11 % (< 96,72 mmol/mol)

Exclusion criteria

Physical or psychological disease likely to interfere with an appropriate conduct of the study

Current drug therapy knowing to interfere with glucose metabolism

Non type 1 diabetes mellitus

Power calculation:

Not applicable as this is a feasibility study

Principal investigators

- Carine de Beaufort Co principal investigator DECCP
- Ulrike Schierloh Co principal investigator DECCP
- Roman Hovorka Co-investigator
- Gosia Wilinska Co-investigator
- Study Sponsor Spidiman project EU

**Introduction**

Type 1 diabetes mellitus is a chronic disease frequently starting in childhood. (1) Good metabolic control is needed from diagnosis onwards to prevent long-term complications. To achieve a good metabolic control necessitates intensive education, frequent insulin injections and frequent blood glucose controls as well as a careful evaluation of food intake and physical activity. This comprehensive day-to-day management and life long task is challenging for the child and its family and has a major impact on their quality of life. (2, 3)

Hyperglycaemia is the major risk factor for micro and macroangiopathy later in life, stressing the importance of obtaining as fast as possible near normoglycaemia (4).

For a long time the improved metabolic control has been associated with an increase in major hypoglycaemic events. More recent studies suggest that with current education, insulins and insulin pumps a good metabolic control can be obtained without major risk for hypoglycaemic events (5-7). It is evident, however, that once a major hypoglycaemic event has been observed this creates a major anxiety in family and patients, frequently leading to a persistent metabolic deterioration. (8)

Short term and long term complications show the need to improve insulin delivery and glucose control. Continuous subcutaneous insulin infusion (CSII) devices, uploaded with continuous glucose monitoring devices (CGM) are now developed to improve metabolic outcome without further increase of the major burden of child and family (9).

Algorithms either based on mathematical modelling PID (Proportional Integral Derivative), MPC (Model predictive control) or MD fuzzy logic may offer the potential to create an Artificial Pancreas (AP), an automated insulin administration based on the information of CGM (10-12) . Through this technology it should be possible to improve metabolic outcome, without hypoglycaemia and without increased burden for families. First results of this are currently becoming available (13)

We have demonstrated the effect of the sensor-uploaded pump, which still needs patient/parent intervention. In the SWITCH study we could demonstrate a reduced time, spent in hypoglycaemia and a reduction in HbA1c when the sensor was used more than 70 % of the time. (14)

Several studies evaluating the outcome of CSII shows a beneficial impact on quality of control as well as on quality of life. (15,16) Although the sensor uploaded pump was expected to lead to further improvement, this has not (yet) been confirmed with the currently used technology. In the younger child, the presence of two injection sites has been mentioned as a possible contributing negative factor. The development of a single port administration of insulin in combination with glucose measurement through one needle/catheter may be a way forward.

Constant presence and information on the glucose values may as well create extra stress and frustration. This continuous confrontation with the diabetes and the continuous necessity to react to the information provided by the sensor has been suggested to increase the burden and contribute to reduced quality of life.

These observations all point towards the need to move to closed loop systems in which intervention of parents/patients is not requested all the time.

Relevant for the development and use of overnight closed loop systems are its different components, the insulin delivery system, the sensor and its reliable feedback as well as the algorithms, regulating insulin delivery.

CSII (insulin delivery systems) as well as the CGM (Continuous Glucose Monitoring) are currently on the market. Further improvement of this will be obtained in phase two (WP9) of the Spidiman project in which a single port entry will be developed and tested (allowing insulin administration and glucose sensing within 1 needle/catheter).

The sensors, used in this study, are both approved for use in the target group.

Recent studies suggest a potential interaction in some cases with different drugs. (17) This potential effect will be evaluated by documenting the drugs used just before and during the study.

Potential changes in measurements of sensors may as well be caused by the local immune reactions, as well as possible immune stimulation through the gut flora. (18)

One of the major contributions to develop a closed-loop system prototype device has been provided by the research team , Cambridge, UK, led by Dr Hovorka, involved in developing novel Model Predictive Controller (MPC) computer algorithms and research into closed-loop insulin delivery since late 1990s (19-21).

The Model Predictive Control (MPC) algorithm estimates patient-specific parameters from CGM glucose measurements taken every 1 to 15 minutes, makes predictions of glucose excursion and calculates basal insulin infusion rates. The MPC approach can handle delays associated with insulin absorption and takes into account meal intake and prandial boluses delivered manually by the patient. The model representation enables simulation of ‘what if’ scenarios, in particular the prediction of future glucose excursions resulting from projected and past insulin infusion rates.

Overnight closed-loop research conducted in Cambridge, UK has shown significantly better performance with closed-loop insulin delivery compared to usual pump therapy in both children and adults (19,21,22). Meta-analysis of these studies in 17 children and adolescents [13.4 ± 3.6 years] and 24 adults (37.5 ± 9.1 years) on 45 closed-loop (intervention) and 45 usual treatment (insulin pump therapy) visits, shows closed-loop increased the time in target plasma glucose in both children and adolescents (from 40% to 60%, p = .002) and adults (from 50% to 76%, p < .001) compared with conventional insulin pump therapy (23). Additionally, closed-loop reduced the time spent below 3.91 mmol/L and above 8.0 mmol/L, from 4.1% to 2.1% (p = .01) and 33% to 20% (p = .03), respectively. Glycaemic variability, as measured by the SD of plasma glucose, was lower during closed-loop compared with CSII (1.5 versus 2.1 mmol/litre, p = .007).

In this project we propose to analyse these developed algorithms overnight in children between 6-12 years.

FlorenceD2 system

The system is purpose-built and comprises a computational device containing the algorithm and communicating with the study CGM device and the study insulin pump.

The closed-loop system comprises a FreeStyle Navigator II® Continuous Glucose Monitoring (CGM) System (Abbott Diabetes Care, Alameda, CA, USA), DANA Diabecare R subcutaneous insulin pump (SOOIL Development co, Seoul, Korea), and a MPC-based glucose control algorithm running on a laptop PC. The FreeStyle Navigator II® CGM device and DANA Diabecare R subcutaneous insulin pump are CE-marked as stand-alone medical devices. An overview of this proposed automated closed-loop system is given in Figure 1

Figure 1: FlorenceD2 closed-loop system

Portable computer running the MPC algorithm


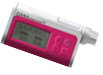

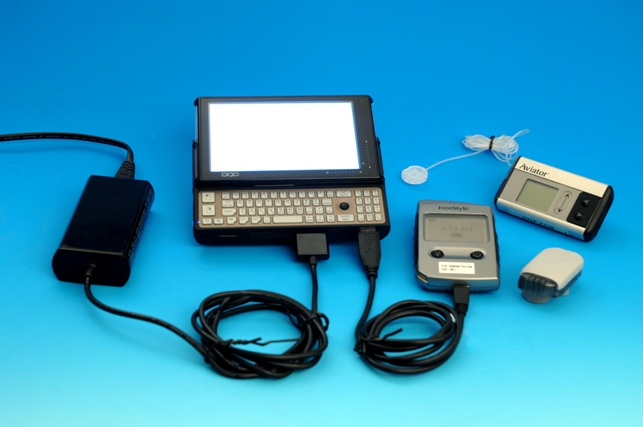


Dana insulin pump

Navigator II Transmitter

Navigator Companion

Navigator Companion

Navigator II Receiver

USB cable

Power cable

A second CGM will be used, but not for insulin administration. It is not linked through telecommunication to the controller, but will serve as objective (called secondary) glucose sensor data source and as extra surveillance during the closed loop at home. It will allow as well a comparison with the blood glucose and insulin measurements and observed insulin delivery , which will serve for optimalisation of the algorithms in this age group.

**New in this study**

- Evaluation of the overnight closed loop algorithms based on MPC in children between 6-12 years in the inpatient setting and at home in a cross over design.
- Collection of glucose, insulin values to validate and improve the algorithms for insulin delivery for this age group.
- Collect faeces to describe the gut flora and its possible interaction with sensor functioning.

**Study design and Patient population**

**Design**

Randomised, 2 period cross over, open label trial to assess efficacy and safety of overnight closed-loop control to target insulin delivery versus open loop insulin delivery in children -with type 1 diabetes aged 6 to 12 years in the inpatient setting and at home. Patients will be randomized in equal numbers to one of the two sequences, (group A or B), which differ only with respect to the initial treatment with (A), or without (B) the overnight closed loop. (Figure 2)

Figure 2

**Patients**

Fifteen Children (to get evaluable data from 12) will be invited to participate with parental informed consent.

Inclusion criteria

- Between 6 - ≤12 years
- With type 1 diabetes for 6 months or more and
- Who use a pump for at least 6 months
- HbA1 should be <11 %.
- No medication or physical or psychological disease should be present, which could interfere with the study.

Written informed consent will be obtained (annex 1)

**Protocol**

Details about the visits and sampling procedure are summarised in table 1a and 1b.

After explanations and consent at visit 1 , the child will be randomised to start either with (group A) or without (group B) closed loop during the inpatient stay and 1 successive night at home.

All children will be invited twice for an overnight stay, once with and once without the closed loop system. During both overnight sessions blood samples will be taken for glucose and insulin measurements, to be used for further testing/improvement of algorithms requested for closing the loop by the Cambridge team.

At visit 2 around two weeks before these overnight stays, the family and patient are invited for a training session in the paediatric clinic. During this session, the DANA pump will be explained. Carb counting as well as technical knowledge on the use of this specific pump will be evaluated before going home.

When sufficient knowledge is acquired, the child will change towards the DANA pump ®. In the same session, we will start with a sensor freestyle Navigator

(Abbott ® ).

During the next two weeks prior to the overnight stay, diabetes control will be as usual , but with sensor and study pump .

Sufficient material will be either provided or prescribed (no extra cost for parents nor CNS)

Visit 3 (the day before the overnight stay in the clinic) will be at the clinic or at home. A new sensor (Navigator) will be placed. A second sensor (Dexcom G4 platinum) will be inserted to validate the Navigator glucose measurements, either at home or in the clinic. This sensor DEXCOM will need initial calibration 2 hrs later, requesting its placement before 1800. This visit will be used to evaluate the appropriate use of pump and sensor.

Visit 4 is the inpatient visit, where at Friday around 1600, the patient and parent(s) will come to the research ward.

The insulin catheter will be changed.

A local anaesthetic cream will be applied before the indwelling cannula is inserted for glucose and insulin measurements.

Blood samples will be taken according to the table 2b.

Stool samples will be collected, when produced, and directly frozen in -80°C (dry ice), or cryoshipper and will be transferred to the IBBL, for storage till measurement.

During the stay in the clinic a meal (content and carb will be decided before to be given at both hospital stays) will be provided in the ward and insulin bolus administration will be done by the parent according to their personal schedule, using the bolus wizard and before the meal. After the meal/bolus the **FlorenceD2 system** will be started in the group with **FlorenceD2 system on**, whereas in the other group their usual nocturnal insulin delivery will be given. A specialised nurse and /or physician will be present during these overnight stays to solve potential questions/problems.

Around 0700 breakfast is planned and in the **FlorenceD2 system on** group, the closed loop control is stopped, and the insulin bolus based on the personal bolus wizard is given followed by breakfast.

For the FlorenceD2 system **off** group, the insulin bolus is given based on the personal bolus wizard, followed by breakfast. Frequent sampling will be continued till 0930 (table 1b, pg16).

Around that time the iv cannula will be removed and the families will go home.

Sensors and pump treatment are maintained and in those randomised for overnight start, the family is invited to use the FlorenceD2 system again at home overnight after the evening bolus and till the next morning before the breakfast bolus. For any problems the dedicated diabetes hotline will be available 24/24 hrs. Training on FlorenceD2 will be provided on Visit 4.

At visit 5 (the Sunday, 24-36 hrs after the overnight stay) the pump catheter will be changed (every 3 days) , the CGM data will be downloaded, the DEXCOM sensor will be removed and one sensor ( Navigator) will not be changed . The FlorenceD2 system will be returned to the clinic and downloaded as well.

Insulin catheter will be changed again on Tuesday/Wednesday and Friday.

During the week the sensors will stay till the Thursday, day when the sensor will be changed. At visit 6, Thursday, (the day before the second overnight stay) , either at home or in the clinic the new sensors will be placed after EMLA.

Visit 7 , on Friday , will be once again in the clinic with an overnight stay with again either closed loop with sampling or control with sampling , and the same protocol will be applied as on visit 4 ( only an exchange of on- off).

The Saturday around 09.30 the family will go home again and use after dinner with the normal bolus the on or off FlorenceD2 system. On Sunday the study will be finished and a final visit (8) will take place in the clinic (or potentially at home). All devices will be returned and the pump catheter will be changed.

Downloads of sensors and pump will be performed in the clinic with the usual clinic technology. Depersonalised, encrypted downloads in ZIP format will be downloaded and used to analyse data. The questionnaires and CRF will stay in Luxembourg at the study center.

Blood samples will be taken according to the sample schedule. For insulin measurements the plasma will be stored till measurement at -20°C.

Venous whole blood Glucose will be measured using the I-Stat (, Glucose oxidase method ABBOTT ®) reporting a CV at 41 mg/dl of 1.6 % and 0.8 % in the higher range (289 mg/dl). Venous samples are around 7 mg/dl lower than capillary ones.

Venous plasma Insulin will be measured by an immunochemiluminometric assay ( invitron , Monmouth, UK) intra assay CV 4.7 % , inter assay CV 7,2-8,1%.

Faeces samples will be frozen, kept at IBBL till analysis. (24). Analysis will include high throughput sequencing.

During the whole study a dedicated and trained team will be available either in person or by phone.

Statistical analysis

A repeated measures regression model with an autoregressive first-order covariance structure adjusted for the period effect, and adjusted for glucose at 21:00, and based on the ranked normal transformation (if endpoints are not normally distributed) will be fit to compare the two treatments.

References

1   Craig M, Hattersley A, Donaghue K. Definition, epidemiology, diagnosis and classification . Pediatric Diabetes 2009 S12; 10: 3-12.

2 [Whittemore R](http://www.ncbi.nlm.nih.gov/pubmed?term=Whittemore R%5BAuthor%5D&cauthor=true&cauthor_uid=22581804), [Jaser S](http://www.ncbi.nlm.nih.gov/pubmed?term=Jaser S%5BAuthor%5D&cauthor=true&cauthor_uid=22581804), [Chao A](http://www.ncbi.nlm.nih.gov/pubmed?term=Chao A%5BAuthor%5D&cauthor=true&cauthor_uid=22581804), [Jang M](http://www.ncbi.nlm.nih.gov/pubmed?term=Jang M%5BAuthor%5D&cauthor=true&cauthor_uid=22581804), [Grey M](http://www.ncbi.nlm.nih.gov/pubmed?term=Grey M%5BAuthor%5D&cauthor=true&cauthor_uid=22581804).

Psychological experience of parents of children with type 1 diabetes: a systematic mixed-studies review. Diabetes Educ. 2012;38(4):562-79

3 Delamater A Psychological care of children and adolescents with diabetes . Ped diabetes 2009 S12 10 175-184

4  Donaghue K, Chiarelli F, Trotta D, Allgrove J, Dahl-Jorgensen K. Microvascular and macrovascular complications Pediatric Diabetes 2009 S12; 10:195-203.

5 [Cengiz E](http://www.ncbi.nlm.nih.gov/pubmed?term=Cengiz E%5BAuthor%5D&cauthor=true&cauthor_uid=23469984), [Xing D](http://www.ncbi.nlm.nih.gov/pubmed?term=Xing D%5BAuthor%5D&cauthor=true&cauthor_uid=23469984), [Wong JC](http://www.ncbi.nlm.nih.gov/pubmed?term=Wong JC%5BAuthor%5D&cauthor=true&cauthor_uid=23469984), [Wolfsdorf JI](http://www.ncbi.nlm.nih.gov/pubmed?term=Wolfsdorf JI%5BAuthor%5D&cauthor=true&cauthor_uid=23469984), [Haymond MW](http://www.ncbi.nlm.nih.gov/pubmed?term=Haymond MW%5BAuthor%5D&cauthor=true&cauthor_uid=23469984), [Rewers A](http://www.ncbi.nlm.nih.gov/pubmed?term=Rewers A%5BAuthor%5D&cauthor=true&cauthor_uid=23469984), [Shanmugham S](http://www.ncbi.nlm.nih.gov/pubmed?term=Shanmugham S%5BAuthor%5D&cauthor=true&cauthor_uid=23469984), [Tamborlane WV](http://www.ncbi.nlm.nih.gov/pubmed?term=Tamborlane WV%5BAuthor%5D&cauthor=true&cauthor_uid=23469984), [Willi SM](http://www.ncbi.nlm.nih.gov/pubmed?term=Willi SM%5BAuthor%5D&cauthor=true&cauthor_uid=23469984), [Seiple DL](http://www.ncbi.nlm.nih.gov/pubmed?term=Seiple DL%5BAuthor%5D&cauthor=true&cauthor_uid=23469984), [Miller KM](http://www.ncbi.nlm.nih.gov/pubmed?term=Miller KM%5BAuthor%5D&cauthor=true&cauthor_uid=23469984), [Dubose SN](http://www.ncbi.nlm.nih.gov/pubmed?term=Dubose SN%5BAuthor%5D&cauthor=true&cauthor_uid=23469984), [Beck RW](http://www.ncbi.nlm.nih.gov/pubmed?term=Beck RW%5BAuthor%5D&cauthor=true&cauthor_uid=23469984); [for the T1D Exchange Clinic Network](http://www.ncbi.nlm.nih.gov/pubmed?term=for the T1D Exchange Clinic Network%5BCorporate Author%5D). Severe hypoglycemia and diabetic ketoacidosis among youth with type 1 diabetes in the T1D Exchange clinic registry. Pediatr Diabetes. 2013 epub

6 Hanas R, Adolfsson P. Insulin pumps in pediatric routine care improve long-term metabolic control without increasing the risk of hypoglycemia. Ped Diabetes. 2006;7:25–31

7 Phillip M, Battelino T, Rodriguez H, Danne T, Kaufman F; European Society for Paediatric Endocrinology; Lawson Wilkins Pediatric Endocrine Society; International Society for Pediatric and Adolescent Diabetes; American Diabetes Association; European Association for the Study of Diabetes. U[se of insulin pump therapy in the pediatric age-group: consensus statement from the European Society for Paediatric Endocrinology, the Lawson Wilkins Pediatric Endocrine Society, and the International Society for Pediatric and Adolescent Diabetes, endorsed by the American Diabetes Association and the European Association for the Study of Diabetes.](http://www.ncbi.nlm.nih.gov/pubmed/17372151)

Diabetes Care. 2007; 30:1653-62

8 Barnard K, Thomas S, Royle P, et al. Fear of hypoglycemia in parents of young children with type 1 diabetes: a systematic review. BMC Pediatr.2010; 10: 50 - 60

9 [Peyrot M](http://www.ncbi.nlm.nih.gov/pubmed?term=Peyrot M%5BAuthor%5D&cauthor=true&cauthor_uid=23496302), [Rubin RR](http://www.ncbi.nlm.nih.gov/pubmed?term=Rubin RR%5BAuthor%5D&cauthor=true&cauthor_uid=23496302); [STAR 3 Study Group](http://www.ncbi.nlm.nih.gov/pubmed?term=STAR 3 Study Group%5BCorporate Author%5D) . Treatment satisfaction in the sensor-augmented pump therapy for A1C reduction 3 (STAR 3) trial. Diabet Med. 2013 30 :464-7.

10 [Mauseth R](http://www.ncbi.nlm.nih.gov/pubmed?term=Mauseth R%5BAuthor%5D&cauthor=true&cauthor_uid=20663457), [Wang Y](http://www.ncbi.nlm.nih.gov/pubmed?term=Wang Y%5BAuthor%5D&cauthor=true&cauthor_uid=20663457), [Dassau E](http://www.ncbi.nlm.nih.gov/pubmed?term=Dassau E%5BAuthor%5D&cauthor=true&cauthor_uid=20663457), [Kircher R Jr](http://www.ncbi.nlm.nih.gov/pubmed?term=Kircher R Jr%5BAuthor%5D&cauthor=true&cauthor_uid=20663457), [Matheson D](http://www.ncbi.nlm.nih.gov/pubmed?term=Matheson D%5BAuthor%5D&cauthor=true&cauthor_uid=20663457), [Zisser H](http://www.ncbi.nlm.nih.gov/pubmed?term=Zisser H%5BAuthor%5D&cauthor=true&cauthor_uid=20663457), [Jovanovic L](http://www.ncbi.nlm.nih.gov/pubmed?term=Jovanovic L%5BAuthor%5D&cauthor=true&cauthor_uid=20663457), [Doyle FJ 3rd](http://www.ncbi.nlm.nih.gov/pubmed?term=Doyle FJ 3rd%5BAuthor%5D&cauthor=true&cauthor_uid=20663457) Proposed clinical application for tuning fuzzy logic controller of artificial pancreas utilizing a personalization factor. J Diabetes Sci Technol. 2010 Jul 1;4(4):913-22

11 Magni L, Raimondo DM, Bossi L, Man CD, De Nicolao G,

Kovatchev B, Cobelli C. [Model predictive control of type 1 diabetes: an in silico trial.](http://www.ncbi.nlm.nih.gov/pubmed/19885152)

J Diabetes Sci Technol. 2007 1(6):804-12.

12 Percival MW, Zisser H, Jovanovic L, Doyle FJ 3rd. [Closed-loop control and advisory mode evaluation of an artificial pancreatic Beta cell: use of proportional-integral-derivative equivalent model-based controllers.](http://www.ncbi.nlm.nih.gov/pubmed/19885240) J Diabetes Sci Technol. 2008;2(4):636-44.

13 Hovorka R, Kumareswaran K, Harris J, Allen JM, Elleri D, Xing D, Kollman C, Nodale M, Murphy HR, Dunger DB, Amiel SA, Heller SR, Wilinska ME, Evans ML. [Overnight closed loop insulin delivery (artificial pancreas) in adults with type 1 diabetes: crossover randomised controlled studies.](http://www.ncbi.nlm.nih.gov/pubmed/21493665)

BMJ. 2011 13;342

14 Battelino T, Conget I, Olsen B, Schütz-Fuhrmann I, Hommel E, Hoogma R, Schierloh U, Sulli N, Bolinder J; SWITCH Study Group. The use and efficacy of continuous glucose monitoring in type 1 diabetes treated with insulin pump therapy: a randomised controlled trial . Diabetologia; 2012 : 55 :3155-62

15 [Cummins E](http://www.ncbi.nlm.nih.gov/pubmed?term=Cummins E%5BAuthor%5D&cauthor=true&cauthor_uid=20223123), [Royle P](http://www.ncbi.nlm.nih.gov/pubmed?term=Royle P%5BAuthor%5D&cauthor=true&cauthor_uid=20223123), [Snaith A](http://www.ncbi.nlm.nih.gov/pubmed?term=Snaith A%5BAuthor%5D&cauthor=true&cauthor_uid=20223123), [Greene A](http://www.ncbi.nlm.nih.gov/pubmed?term=Greene A%5BAuthor%5D&cauthor=true&cauthor_uid=20223123), [Robertson L](http://www.ncbi.nlm.nih.gov/pubmed?term=Robertson L%5BAuthor%5D&cauthor=true&cauthor_uid=20223123), [McIntyre L](http://www.ncbi.nlm.nih.gov/pubmed?term=McIntyre L%5BAuthor%5D&cauthor=true&cauthor_uid=20223123), [Waugh N](http://www.ncbi.nlm.nih.gov/pubmed?term=Waugh N%5BAuthor%5D&cauthor=true&cauthor_uid=20223123).

Clinical effectiveness and cost-effectiveness of continuous subcutaneous insulin infusion for diabetes: systematic review and economic evaluation. Health Technol Assess. 2010 14(11): 1-181.

16. [McMahon SK](http://www.ncbi.nlm.nih.gov/pubmed?term=McMahon SK%5BAuthor%5D&cauthor=true&cauthor_uid=15606698), [Airey FL](http://www.ncbi.nlm.nih.gov/pubmed?term=Airey FL%5BAuthor%5D&cauthor=true&cauthor_uid=15606698), [Marangou DA](http://www.ncbi.nlm.nih.gov/pubmed?term=Marangou DA%5BAuthor%5D&cauthor=true&cauthor_uid=15606698), [McElwee KJ](http://www.ncbi.nlm.nih.gov/pubmed?term=McElwee KJ%5BAuthor%5D&cauthor=true&cauthor_uid=15606698), [Carne CL](http://www.ncbi.nlm.nih.gov/pubmed?term=Carne CL%5BAuthor%5D&cauthor=true&cauthor_uid=15606698), [Clarey AJ](http://www.ncbi.nlm.nih.gov/pubmed?term=Clarey AJ%5BAuthor%5D&cauthor=true&cauthor_uid=15606698), [Davis EA](http://www.ncbi.nlm.nih.gov/pubmed?term=Davis EA%5BAuthor%5D&cauthor=true&cauthor_uid=15606698), [Jones TW](http://www.ncbi.nlm.nih.gov/pubmed?term=Jones TW%5BAuthor%5D&cauthor=true&cauthor_uid=15606698).Insulin pump therapy in children and adolescents: improvements in key parameters of diabetes management including quality of life. Diabet Med. 2005;22(1):92-6

17 Klueh U, Kaur M, Qiao Y, Kreutzer DL [Critical role of tissue mast cells in controlling long-term glucose sensor function in vivo.](http://www.ncbi.nlm.nih.gov/pubmed/20226521).Biomaterials. 2010 31(16):4540-51

18 Sheth D, Natarajab P , Donovan J , Li X, Schneider B , Isayeva I. In vitro drug interference with electrochemical enzymatic glucose sensors abstracts meeting FDA NIH Wastington 2013

19 [Hovorka R](http://www.ncbi.nlm.nih.gov/pubmed?term=Hovorka R%5BAuthor%5D&cauthor=true&cauthor_uid=20138357), [Allen JM](http://www.ncbi.nlm.nih.gov/pubmed?term=Allen JM%5BAuthor%5D&cauthor=true&cauthor_uid=20138357), [Elleri D](http://www.ncbi.nlm.nih.gov/pubmed?term=Elleri D%5BAuthor%5D&cauthor=true&cauthor_uid=20138357), [Chassin LJ](http://www.ncbi.nlm.nih.gov/pubmed?term=Chassin LJ%5BAuthor%5D&cauthor=true&cauthor_uid=20138357), [Harris J](http://www.ncbi.nlm.nih.gov/pubmed?term=Harris J%5BAuthor%5D&cauthor=true&cauthor_uid=20138357), [Xing D](http://www.ncbi.nlm.nih.gov/pubmed?term=Xing D%5BAuthor%5D&cauthor=true&cauthor_uid=20138357), [Kollman C](http://www.ncbi.nlm.nih.gov/pubmed?term=Kollman C%5BAuthor%5D&cauthor=true&cauthor_uid=20138357), [Hovorka T](http://www.ncbi.nlm.nih.gov/pubmed?term=Hovorka T%5BAuthor%5D&cauthor=true&cauthor_uid=20138357), [Larsen AM](http://www.ncbi.nlm.nih.gov/pubmed?term=Larsen AM%5BAuthor%5D&cauthor=true&cauthor_uid=20138357), [Nodale M](http://www.ncbi.nlm.nih.gov/pubmed?term=Nodale M%5BAuthor%5D&cauthor=true&cauthor_uid=20138357), [De Palma A](http://www.ncbi.nlm.nih.gov/pubmed?term=De Palma A%5BAuthor%5D&cauthor=true&cauthor_uid=20138357), [Wilinska ME](http://www.ncbi.nlm.nih.gov/pubmed?term=Wilinska ME%5BAuthor%5D&cauthor=true&cauthor_uid=20138357), [Acerini CL](http://www.ncbi.nlm.nih.gov/pubmed?term=Acerini CL%5BAuthor%5D&cauthor=true&cauthor_uid=20138357), [Dunger DB](http://www.ncbi.nlm.nih.gov/pubmed?term=Dunger DB%5BAuthor%5D&cauthor=true&cauthor_uid=20138357). Manual closed-loop insulin delivery in children and adolescents with type 1 diabetes: a phase 2 randomised crossover trial. Lancet. 2010 375 :743-51.

20 [Hovorka R](http://www.ncbi.nlm.nih.gov/pubmed?term=Hovorka R%5BAuthor%5D&cauthor=true&cauthor_uid=15198833), [Chassin LJ](http://www.ncbi.nlm.nih.gov/pubmed?term=Chassin LJ%5BAuthor%5D&cauthor=true&cauthor_uid=15198833), [Wilinska ME](http://www.ncbi.nlm.nih.gov/pubmed?term=Wilinska ME%5BAuthor%5D&cauthor=true&cauthor_uid=15198833), [Canonico V](http://www.ncbi.nlm.nih.gov/pubmed?term=Canonico V%5BAuthor%5D&cauthor=true&cauthor_uid=15198833), [Akwi JA](http://www.ncbi.nlm.nih.gov/pubmed?term=Akwi JA%5BAuthor%5D&cauthor=true&cauthor_uid=15198833), [Federici MO](http://www.ncbi.nlm.nih.gov/pubmed?term=Federici MO%5BAuthor%5D&cauthor=true&cauthor_uid=15198833), [Massi-Benedetti M](http://www.ncbi.nlm.nih.gov/pubmed?term=Massi-Benedetti M%5BAuthor%5D&cauthor=true&cauthor_uid=15198833), [Hutzli I](http://www.ncbi.nlm.nih.gov/pubmed?term=Hutzli I%5BAuthor%5D&cauthor=true&cauthor_uid=15198833), [Zaugg C](http://www.ncbi.nlm.nih.gov/pubmed?term=Zaugg C%5BAuthor%5D&cauthor=true&cauthor_uid=15198833), [Kaufmann H](http://www.ncbi.nlm.nih.gov/pubmed?term=Kaufmann H%5BAuthor%5D&cauthor=true&cauthor_uid=15198833), [Both M](http://www.ncbi.nlm.nih.gov/pubmed?term=Both M%5BAuthor%5D&cauthor=true&cauthor_uid=15198833), [Vering T](http://www.ncbi.nlm.nih.gov/pubmed?term=Vering T%5BAuthor%5D&cauthor=true&cauthor_uid=15198833), [Schaller HC](http://www.ncbi.nlm.nih.gov/pubmed?term=Schaller HC%5BAuthor%5D&cauthor=true&cauthor_uid=15198833), [Schaupp L](http://www.ncbi.nlm.nih.gov/pubmed?term=Schaupp L%5BAuthor%5D&cauthor=true&cauthor_uid=15198833), [Bodenlenz M](http://www.ncbi.nlm.nih.gov/pubmed?term=Bodenlenz M%5BAuthor%5D&cauthor=true&cauthor_uid=15198833), [Pieber TR](http://www.ncbi.nlm.nih.gov/pubmed?term=Pieber TR%5BAuthor%5D&cauthor=true&cauthor_uid=15198833). Closing the loop: the adicol experience. Diabetes Technol Ther. 2004;6(3):307-18.

21 [Hovorka R](http://www.ncbi.nlm.nih.gov/pubmed?term=Hovorka R%5BAuthor%5D&cauthor=true&cauthor_uid=21493665), [Kumareswaran K](http://www.ncbi.nlm.nih.gov/pubmed?term=Kumareswaran K%5BAuthor%5D&cauthor=true&cauthor_uid=21493665), [Harris J](http://www.ncbi.nlm.nih.gov/pubmed?term=Harris J%5BAuthor%5D&cauthor=true&cauthor_uid=21493665), [Allen JM](http://www.ncbi.nlm.nih.gov/pubmed?term=Allen JM%5BAuthor%5D&cauthor=true&cauthor_uid=21493665), [Elleri D](http://www.ncbi.nlm.nih.gov/pubmed?term=Elleri D%5BAuthor%5D&cauthor=true&cauthor_uid=21493665), [Xing D](http://www.ncbi.nlm.nih.gov/pubmed?term=Xing D%5BAuthor%5D&cauthor=true&cauthor_uid=21493665), [Kollman C](http://www.ncbi.nlm.nih.gov/pubmed?term=Kollman C%5BAuthor%5D&cauthor=true&cauthor_uid=21493665), [Nodale M](http://www.ncbi.nlm.nih.gov/pubmed?term=Nodale M%5BAuthor%5D&cauthor=true&cauthor_uid=21493665), [Murphy HR](http://www.ncbi.nlm.nih.gov/pubmed?term=Murphy HR%5BAuthor%5D&cauthor=true&cauthor_uid=21493665), [Dunger DB](http://www.ncbi.nlm.nih.gov/pubmed?term=Dunger DB%5BAuthor%5D&cauthor=true&cauthor_uid=21493665), [Amiel SA](http://www.ncbi.nlm.nih.gov/pubmed?term=Amiel SA%5BAuthor%5D&cauthor=true&cauthor_uid=21493665), [Heller SR](http://www.ncbi.nlm.nih.gov/pubmed?term=Heller SR%5BAuthor%5D&cauthor=true&cauthor_uid=21493665), [Wilinska ME](http://www.ncbi.nlm.nih.gov/pubmed?term=Wilinska ME%5BAuthor%5D&cauthor=true&cauthor_uid=21493665), [Evans ML](http://www.ncbi.nlm.nih.gov/pubmed?term=Evans ML%5BAuthor%5D&cauthor=true&cauthor_uid=21493665). Overnight closed loop insulin delivery (artificial pancreas) in adults with type 1 diabetes: crossover randomised controlled studies. BMJ. 2011 342:

22 [.](http://www.ncbi.nlm.nih.gov/pubmed/21355719) Elleri D, Allen JM, Nodale M, Wilinska ME, Mangat JS, Larsen AM, Acerini CL, Dunger DB, Hovorka R. Automated overnight closed-loop glucose control in young children with type 1 diabetes

Diabetes Technol Ther. 2011 13(4):419-24

23 Kumareswaran K, Elleri D, Allen JM, Harris J, Xing D, Kollman C, Nodale M, Murphy HR, Amiel SA, Heller SR, Wilinska ME, Acerini CL, Evans ML, Dunger DB, Hovorka R.

[Meta-analysis of overnight closed-loop randomized studies in children and adults with type 1 diabetes: the Cambridge cohort.](http://www.ncbi.nlm.nih.gov/pubmed/22226252)

J Diabetes Sci Technol. 2011 ;5 (6):1352-62.

24 [Roume H](http://www.ncbi.nlm.nih.gov/pubmed?term=Roume H%5BAuthor%5D&cauthor=true&cauthor_uid=22763648), [Muller EE](http://www.ncbi.nlm.nih.gov/pubmed?term=Muller EE%5BAuthor%5D&cauthor=true&cauthor_uid=22763648), [Cordes T](http://www.ncbi.nlm.nih.gov/pubmed?term=Cordes T%5BAuthor%5D&cauthor=true&cauthor_uid=22763648), [Renaut J](http://www.ncbi.nlm.nih.gov/pubmed?term=Renaut J%5BAuthor%5D&cauthor=true&cauthor_uid=22763648), [Hiller K](http://www.ncbi.nlm.nih.gov/pubmed?term=Hiller K%5BAuthor%5D&cauthor=true&cauthor_uid=22763648), [Wilmes P](http://www.ncbi.nlm.nih.gov/pubmed?term=Wilmes P%5BAuthor%5D&cauthor=true&cauthor_uid=22763648)

A biomolecular isolation framework for eco-systems biology. ISME J. 2013 Jan;7(1):110-21. doi: 10.1038/ismej.2012.72. Epub 2012 Jul 5.

Table 1a Description of the visits

*d 0-7 will be a Thursday

° download before departure at home

. .

**Visits time Action Questionnaires/Downloads**

V1 Detailed Information on protocol

Consent and randomisation CRF1

V2 -2 wks Training in the outpatient clinic to use the DANA CRF2

pump FFQ

Training to use the sensor Navigator Competency assessment

V3 d0* Change of sensor and adding second sensor, download CSII/CGM

providing EMLA to apply 1,5 hrs prior to the clinic visit V4

V4 d1 Arrival around 1600, Catheter change and

placement of indwelling venous cannula 24hRecall food intake

24h for sampling of insulin – glucose ( details in table 2b) CRF3

Start closed loop (group A) or open loop evaluation (group B)

after dinner (details in table 2b) Download CSII/CGM

When needed explanation to the family the handling of the

closed loop system (A)

d2 Closed loop (A) starting after dinner at home, stop next Download CSII/CGM°

morning before breakfast

V5 d3 Return FlorenceD2 closed loop system material Download CSII/CGM Change catheter CRF3.2

Problems encountered User Quality of Care1 Evaluation

d5 Change catheter at home (D5 or d6) Phone contact/Home/clinic visit

V6 d7* New sensors (dexcom/navigator) Download CSII/CGM

providing EMLA to apply 1,5 hrs prior to the clinic visit V7

V7 d8 Arrival around 1600 , change catheter ,

placement of indwelling venous cannula 24h Recall foodintake

for sampling of insulin – glucose ( details in table 2b) Download CSII/CGM

Start closed loop (B) or open loop (A) evaluation after dinner CRF3.3

(details in table 2b)

When needed explanation to the family the handling of the

closed loop system (B)

d9 Closed loop (B) starting after dinner at home, stop next Download CSII/CGM°  morning before breakfast

V8 d10 Change back to previous system Download CSII/CGM

Return AP CAM material CRF3.4

Discuss Problems encountered User Quality of Care evaluation

. .

Table 1b Sequence of the procedure

| Time | ACTION/meals and snacks | Plasma glucose | Plasma  insulin | CGM | CSII | BOLUS |
| --- | --- | --- | --- | --- | --- | --- |
| 1630 | Arrival at clinic  Canulation |  |  | X | X |  |
| 1700 | Start sampling | x | x | X | x |  |
| 1800 | Meal | x | x | X | x | X |
| 1830 | Start closed loop (A)  explain, training on starting and stopping closed loop start | x | x | X | x |  |
| 1900 | Play | X | x | X | x |  |
| 1930 | Play | x | x | X | x |  |
| 20.00 | Time for bed | x | x | X | x |  |
| 2100 | Lights out | x | x | X | x |  |
| 2200 |  | x | x | X | x |  |
| 2300 |  | x | x | X | x |  |
| 2400 |  | x | x | X | x |  |
| 0100 |  | x | x | X | x |  |
| 0200 |  | X | x | X | x |  |
| 0300 |  | x | x | X | x |  |
| 0400 |  | x | x | X | x |  |
| 0500 |  | x | x | X | x |  |
| 0600 |  | x | x | X | x |  |
| 0700 | Waking up | x | x | X | x |  |
| 0730 | Breakfast, STOP closed loop | x | X | X | x | X |
| 0800 |  | X | X | X | X |  |
| 0830 |  | x | x | X | X |  |
| 0930 | Last control removal cannula | x | x | X | x |  |
|  | HOME |  |  | X | x |  |
|  | During the stay : collect 1 stool sample directly at -80 or in cryoshipper | | | | | |

Blood volume : glucose /insuline : 21 x 1,5 ml (32,5ml)

Biomarker sample 1,5 ml

**Data analysis**

Questionnaire data will be entered on paper and data entry in the data base will be performed in Luxembourg , study center Clinique Pédiatrique de Luxembourg.

The sensor data and pump data will be read through the routine source , used for clinical practice in the CHL. Depersonalised, encrypted downloads in ZIP format will be downloaded and used to analyse data in Cambridge ,UK

Blood glucose and insulin values , will be entered into the data base dedicated for the study and depersonalised transferred to the UK Cambridge group .

In general a 5% significance will be used to declare statistical significance .

Both glucose data obtained through the sensors will be compared with the blood glucose values.

The time in target and the different mentioned indicators will be calculated comparing the data obtained with and without the closed loop in the inpatient and home setting .

A comparison will be made between the data obtained at home and in the clinic .

All blood glucose and sensor data will be analysed anonymously , eg the person analysing the data will be blinded for the type of overnight profile

Finally glucose and insulin values will serve for improved algorithm development by the CAM team, ( prof Hovorka and Wilinska) university of Cambridge, UK

CRF1 Name STUDY number

Written Consent yes ☐ Study number

Gender F ☐ M ☐ Randomisation A☐ B ☐

DoB …./…./….

Date Diagnosis :

Date Start pump treatment PUMP type

Number of Severe hypoglycemic events over the last 3 months ☐☐

Number of severe DKA events over the last 12 months ☐☐

Frequency of BG measurement

Medication during the last 48 hours , other than insulin yes : ☐ no☐

If yes : please specify :

Concomittant disease : coeliac ☐ thyroid ☐ other ☐

Family situation :…………………………..

Ethnicity : Caucasian ☐, Black ☐ , Asian ☐ Hispanic ☐ other

CLINICAL DATA

BW kg Height cm

BP mmHg Normal clinical examination yes ☐ no ☐

If not, please specify

Intercurrent infections : yes ☐ no ☐ ,

If yes , please specify

Pump type

**Pump setting**

Basal rate total ,

hourly

bolus administration 24 h before the visit

Bolus wizard programmation

Ratio insulin carb g/u

Insulin sensibility mg/u

Insulin action duration ..h

Target values …

CRF2 Visit V2

Date NAME STUDY number

Intercurrent infections : yes ☐ no ☐ , if yes , please specify

**Adverse events since the previous visit**

Number of severe hypoglycemic events ( requiring iv glucose /glucagon) ☐ ☐ ☐

Number of Severe ketoacidosis ( requiring iv isulin administration) ☐ ☐ ☐

Pump type

**Pump setting**

Basal rate total ,

hourly

bolus administration 24 h before the visit

Bolus wizard programmation

Ratio insulin carb g/u

Insulin sensibility mg%/U

Insulin action duration ..h

Target values …

Date place skin aspects

Canule site

Sensor Navigator site

Sensor DEXCOM site

PUMP SETTING if changed

CRF3 Visit V4 ☐ V5 ☐ V7 ☐ V8 ☐

Date NAME STUDY number

Intercurrent infections : yes ☐ no ☐ , if yes , please specify

**Adverse events since the previous visit**

Number of Severe Hypoglycemic events ( requiring iv glucose /glucagon) ☐☐☐

Number of Severe Ketoacidosis ( requiring iv isulin administration) ☐☐☐

**Pump setting**

Basal rate total ,

hourly

bolus administration 24 h before the visit

Ratio insulin carb g/u

Insulin sensibility mg/u

Insulin action duration ..h

Target values …

Date place aspect

Canule site

Sensor Navigator site

Sensor DEXCOM site

PUMP SETTING if changed
